# Identification of the protonation and oxidation states of the oxygen-evolving complex in the low-dose X-ray crystal structure of photosystem II

**DOI:** 10.3389/fpls.2023.1029674

**Published:** 2023-03-16

**Authors:** Keisuke Saito, Shu Nakao, Hiroshi Ishikita

**Affiliations:** ^1^ Research Center for Advanced Science and Technology, The University of Tokyo, Tokyo, Japan; ^2^ Department of Applied Chemistry, The University of Tokyo, Tokyo, Japan

**Keywords:** crystal structure, x-ray radiation damage, low barrier hydrogen bond, photosystem II, oxygen evolving complex, quantum mechanics/molecular mechanics (QM/MM)

## Abstract

In photosystem II (PSII), the O3 and O4 sites of the Mn_4_CaO_5_ cluster form hydrogen bonds with D1-His337 and a water molecule (W539), respectively. The low-dose X-ray structure shows that these hydrogen bond distances differ between the two homogeneous monomer units (A and B) [Tanaka et al., J. Am Chem. Soc. 2017, 139, 1718]. We investigated the origin of the differences using a quantum mechanical/molecular mechanical (QM/MM) approach. QM/MM calculations show that the short O4-O_W539_ hydrogen bond (~2.5 Å) of the B monomer is reproduced when O4 is protonated in the S_1_ state. The short O3-Nε_His337_ hydrogen bond of the A monomer is due to the formation of a low-barrier hydrogen bond between O3 and doubly-protonated D1-His337 in the overreduced states (S_−1_ or S_−2_). It seems plausible that the oxidation state differs between the two monomer units in the crystal.

## Introduction

**Figure 1:**
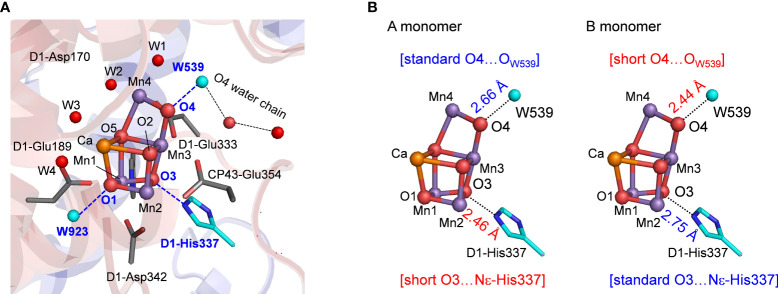
**(A)** Structure and hydrogen-bond partner (cyan) of oxygen atoms of the Mn_4_CaO_5_ cluster. W539 is a part of the O4 water chain that functions as the proton transfer pathway in the proton release during the S_0_ to S_1_ transition. **(B)** Differences in hydrogen-bond distances of the Mn_4_CaO_5_ cluster between the A and B monomers of the low-dose structure (0.03 MGy; PDB-ID 5B5E).

The reaction center in photosystem II (PSII) has the O_2_ evolving complex, Mn_4_CaO_5_ cluster (**Figure 1A**) (Shen, 2015; Cardona and Rutherford, 2019). O_2_ evolves at the Mn_4_CaO_5_ cluster, which has five O atoms (O1 to O5), two ligand water molecules at the Mn4 site (W1 and W2), and two additional water molecules at the Ca site (W3 and W4). O1 and O4 form an hydrogen bond to water molecules (**Figure 1A**). To convert two substrate water molecules into O_2_, four electrons and four protons must be removed. As electron transfer proceeds, the oxidation state of the Mn_4_CaO_5_ cluster, S*_n_*, increases, and protons are released with a typical stoichiometry of 1:0:1:2 for the S_0_ → S_1_ → S_2_ → S_3_ → S_0_ transitions (Suzuki et al., 2005). O_2_ evolves during the S_3_ to S_0_ transition, followed by the first proton release during the S_0_ to S_1_ transition. Based on density functional theory (DFT) calculations performed in the absence of the PSII protein environment, it was proposed that the O5 site was protonated in S_0_ (Siegbahn, 2013; Krewald et al., 2015; Lohmiller et al., 2017). However, the PSII structure shows that O5 has no H-bond partner, which suggests that the release of the proton from O5 is unlikely to occur in the PSII protein environment. In contrast, the O4 site forms a significantly short hydrogen bond (< 2.5 Å) with the adjacent water molecule (W539) in the PSII structures (Umena et al., 2011; Suga et al., 2015). Quantum mechanical/molecular mechanical (QM/MM) calculations indicated that O4 and W539 form a low-barrier hydrogen bond (LBHB), which facilitates the release of the proton from O4 during the S_0_ to S_1_ transition (Saito et al., 2015; Takaoka et al., 2016). Time resolved infrared (TRIR) and Fourier transform infrared (FTIR) studies (Shimizu et al., 2018; Yamamoto et al., 2020) suggested that the S_0_ to S_1_ transition is the fastest among all S state transitions, which is in line with the formation of the LBHB between O4 and W539.

The Mn_4_CaO_5_ cluster structure was determined by X-ray diffraction (XRD) crystallography at a resolution of 1.9 Å (Umena et al., 2011) on the assumption that it was in the dark-stable S_1_ state, as the PSII crystals used for the diffraction experiments were stored in the dark during crystallization and the diffraction intensity measurements. The structure was obtained by using a high-dose X-ray (an average dose of 0.43 MGy) and is referred to as the high-dose structure. Thus, the Mn_4_CaO_5_ cluster may have been overreduced, including Mn(II) (Yano et al., 2005; Grabolle et al., 2006; Luber et al., 2011; Galstyan et al., 2012; Glockner et al., 2013; Saito and Ishikita, 2019). Using X-ray free-electron laser (XFEL), the “radiation-damage-free” structure was reported (Suga et al., 2015). However, it was suggested that the Mn_4_CaO_5_ cluster is reduced to S_0_ even in the XFEL structures (Askerka et al., 2015). Tanaka et al. determined structures using significantly lower X-ray doses (0.03 and 0.12 MGy) with conventional synchrotron radiation sources (low-dose structures) (Tanaka et al., 2017).

**Figure 2:**
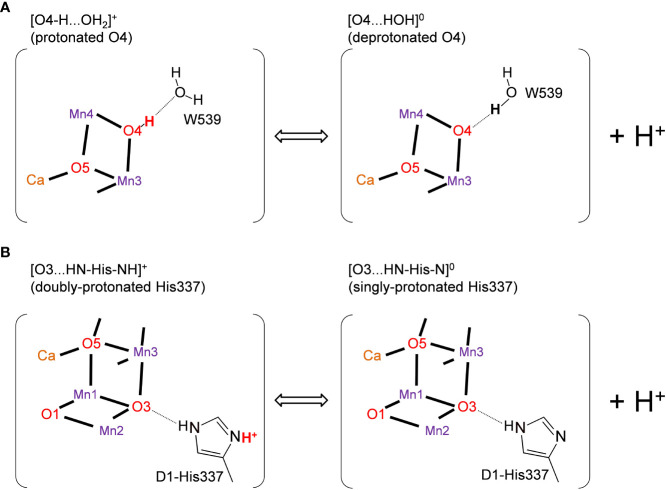
Possible protonation states of **(A)** O4 and **(B)** D1-His337 (Tanaka et al., 2017). The protonated and deprotonated O4 patterns correspond to "pre-PT" and "post-PT" (PT: proton transfer), respectively, in (Saito et al, 2015).

In the low-dose PSII structures, the hydrogen bond distances differ significantly between the two monomer units (A and B) (Tanaka et al., 2017). The main differences were as follows: (1) the hydrogen bond between O3 and D1-His337 was shorter in the A monomer (2.46 Å) than in the B monomer (2.75 Å) (**Figure 1B**); (2) the hydrogen bond between O4 and W539 [W6 in ref. (Tanaka et al., 2017)] was longer in the A monomer (2.66 Å) than in the B monomer (2.44 Å) (**Figure 1B**).

It was speculated that the differences in the hydrogen-bond distance were due to the difference in the protonation states of D1-His337 and W539 (Tanaka et al., 2017). According to Tanaka et al., D1-His337 might be doubly-protonated [HN-His-NH]^+^ in the short O3-His337 hydrogen bond and singly-protonated [HN-His-N]^0^ in the long O3-His337 hydrogen bond (**Figure 2B**). Tanaka et al. also speculated that W539 existed as H_3_O^+^ in the short O4-W539 hydrogen bond. However, H_3_O^+^ (p*K*_a_ = −1.7) can exist only when the binding moiety is stabilized by a cluster of acidic residues (Ikeda et al., 2017). In contrast, there exists the positively charged Mn_4_CaO_5_ cluster adjacent to W539, which inhibits the formation of H_3_O^+^. Thus, either [O4-H...O_W539_H_2_]^+^ (protonated O4) or [O4...HO_W539_H]^0^ (deprotonated O4) can be more relevant (Saito et al., 2015) (**Figure 2A**). The details of the protonation states in the low-dose structure are not reported. Here, we investigate the protonation state in the low-dose structure using a QM/MM approach.

## Methods

The PSII atomic coordinates were taken from the lower dose structure (0.03 MGy; PDB-ID 5B5E) (Tanaka et al., 2017). The H atom positions were optimized with CHARMM (Brooks et al., 1983), whereas the heavy atom positions were fixed. During the procedure, all titratable groups (e.g., acidic and basic groups) were ionized. Additional counter ions were added to neutralize the entire system in QM/MM calculations. Atomic partial charges of the amino acids were obtained from the CHARMM22 (MacKerell et al., 1998) parameter set, whereas those of cofactors were obtained from previous studies (Saito et al., 2015). D1-Asp61 is fully ionized (Ishikita et al., 2006). Note that other titratable residues (e.g., D1-Glu65 and D2-Glu312) are >13 Å away from the Mn_4_CaO_5_ cluster.

The QM/MM calculation was performed as done in previous studies (Mandal et al., 2020). Using the QSite (QSite, 2012) program, the unrestricted DFT method was employed with the B3LYP functional and LACVP** basis sets. The atom positions in the QM region were fully relaxed, whereas the H atom positions in the MM region were optimized using the OPLS2005 force field. The Mn_4_CaO_5_ cluster was considered to be ferromagnetically coupled (i.e., the total spin *S* = 14/2, 15/2, 16/2, and 17/2 in S_1_, S_0_, S_−1_, and S_−2_, respectively). Notably, the resulting optimized Mn_4_CaO_5_ geometry appears not to be crucial to the spin configurations, as demonstrated in previous theoretical studies (Ames et al., 2011; Isobe et al., 2012). Indeed, the calculated distances in the anti-ferromagnetically coupled (i.e. low spin) cases (**Table S1**) were essentially the same as the ferromagnetically coupled (i.e., high spin) cases. The Mn valence states were determined by spin densities obtained from the Mulliken population analysis (**Table S2**). O1, O2, O3, and O5 were considered to be unprotonated (O^2–^), while O4 was considered to be protonated (OH^−^) in the protonated O4 hydrogen-bond pattern (**Figure 2A**).

The initial-guess wavefunctions were obtained using the ligand field theory (Vacek et al., 1999) implemented in the QSite program. The QM region was defined as the Mn_4_CaO_5_ cluster (including the ligand side-chains of D1-Asp170, D1-Glu189, D1-His332, D1-Glu333, D1-Asp342, and CP43-Glu354; the ligand carboxy-terminal group of D1-Ala344; and the ligand water molecules, W1–W4), the O4-water chain (W539, W538, and W393) (Saito et al., 2015; Takaoka et al., 2016), the Cl-1 binding site (Cl-1, W442, W446, and the side-chains of D1-Asn181 and D2-Lys317), the second-sphere ligands (side-chains of D1-Asp61 and CP43-Arg357), and the hydrogen-bond network of TyrZ (side-chains of D1-Tyr161, D1-His190, and D1-Asn298), including the diamond-shaped cluster of water molecules (W5, W6, and W7) (Saito et al., 2011; Kawashima et al., 2018). To calculate the distance of the LBHB in the [O3…H-His-H]^+^ pattern in S_−2_, the QM region was extended to include the hydrogen-bond network of three water molecules near D1-His337. All other protein units and cofactors were approximated by the MM force field. See Supporting Information for the atomic coordinates of the resulting QM region.

## Results and discussion

The O4…O_W539_ hydrogen bond is particularly short in the B monomer of the low-dose structure (2.44 and 2.45 Å in the 0.03 and 0.12 MGy structures, respectively). In the QM/MM geometry optimization using the 0.03 MGy low-dose structure, a short hydrogen bond is reproduced (2.48 and 2.44 Å) when O4 is protonated ([O4-H…O_W539_H_2_]^+^) and D1-His337 is doubly-protonated ([HN-His-NH]^+^) in S_1_ in both the A and B monomers (**Table 1**), as previously reported in a QM/MM study on the high-dose structure (Saito et al., 2015).

The O3…Nε_His337_ hydrogen bond is particularly short in the B monomer of the low-dose structure (2.46 and 2.48 Å in the 0.03 and 0.12 MGy structures, respectively). In the QM/MM geometry optimization, the short hydrogen bond cannot be reproduced regardless of the protonation state of D1-His337 ([HN-His-NH]^+^ and [HN-His]^0^) in both S_0_ and S_1_ (**Table 1**). Notably, the protonation state of O4 did not substantially affect the O3…Nε_His337_ distance. Thus, the short hydrogen bond between O3 and D1-His337 in the A monomer cannot be explained by the difference in the protonation state of D1-His337 in contrast to the mechanism speculated by Tanaka et al. (Tanaka et al., 2017).

**Table 1:** Hydrogen bond distances for the low dose and QM/MM-optimized structures (in Å).

Unit ^a^				A		B	
Structure		D1-His337	O4-W539	O4…O_W539_	O3…Nε_His337_	O4…O_W539_	O3…Nε_His337_
Crystal							
Low-dose							
5B5E (0.03 MGy)				2.66	**2.46**	**2.44**	2.75
5B66 (0.12 MGy)				2.71	**2.48**	**2.45**	2.74
QM/MM							
(Standard oxidation state)							
Low-dose (5B5E)	S_0_	[H-His-H]^+^	[O4-H…OH_2_]^+^ ^b^	2.60	2.66	2.55	2.67
		[His-H]^0^		2.61	2.74	2.57	2.74
		[H-His-H]^+^	[O4…HOH]^0^ ^c^	2.57	2.61	2.56	2.61
		[His-H]^0^		2.55	2.75	2.55	2.73
	S_1_	[H-His-H]^+^	[O4-H…OH_2_]^+^ ^d^	**2.48**	2.72	**2.44**	2.74
		[His-H]^0^		2.51	2.77	**2.46**	2.80
		[H-His-H]^+^	[O4…HOH]^0^ ^d^	2.63	2.67	2.62	2.68
		[His-H]^0^		2.61	2.74	2.61	2.73
(Overreduced state)							
Low-dose (5B5E)	S_−1_	[H-His-H]^+^	[O4-H…OH_2_]^+^ ^e^	2.64	2.58	2.61	2.57
		[His-H]^0^		2.64	2.72	2.63	2.71
		[H-His-H]^+^	[O4…HOH]^0^ ^e^	2.57	2.54	2.57	2.56
		[His-H]^0^		2.61	2.72	2.59	2.71
	S_−2_	[O3-H…His-H]^+^	[O4-H…OH_2_]^+^ ^f^	2.72	2.60	2.67	2.57
		[O3…H-His-H]^+^		2.75	2.56	2.70	**2.53**
		[His-H]^0^		2.76	2.69	2.68	2.68
		[H-His-H]^+^	[O4…HOH]^0^ ^f^	2.52	2.64	―^g^	―^g^
		[His-H]^0^		―^g^	―^g^	―^g^	―^g^

a PSII monomer unit ID in the PSII dimer.

b (Mn1, Mn2, Mn3, Mn4) = (III, IV, III, III).

c (Mn1, Mn2, Mn3, Mn4) = (III, III, IV, III).

d (Mn1, Mn2, Mn3, Mn4) = (III, IV, IV, III).

e (Mn1, Mn2, Mn3, Mn4) = (III, III, III, III).

f (Mn1, Mn2, Mn3, Mn4) = (III, III, III, II).

g S_−2_ was not obtained due to internal electron transfer from Mn4 to D1-Asn298. Short distances (< 2.5 Å for the O-O distance and < 2.55 Å for the O-N distance) are in bold. ―, not applicable. D1-His337 is either doubly-protonated ([H-His-H]^+^) or singly-protonated ([His-H]^0^). O4 is either protonated ([O4-H...OH_2_]^+^) or deprotonated ([O4...HOH]^0^).

A possible reason for the short hydrogen bond between O3 and D1-His337 could be the overreduced state of the Mn_4_CaO_5_ cluster. A DFT study demonstrated that the proton of D1-His337 can be transferred to O3 during overreduction of the Mn_4_CaO_5_ cluster (Galstyan et al., 2012). A previous QM/MM study on the high-dose structure also reported that the hydrogen-bond distance between O3 and D1-His337 decreased (2.58 Å for O3…Nε_His337_) when the Mn_4_CaO_5_ cluster was overreduced in S_−2_ (Saito and Ishikita, 2019). Therefore, the short hydrogen bond of the low-dose structures may originate from the overreduced state in the crystal.

**Figure 3:**
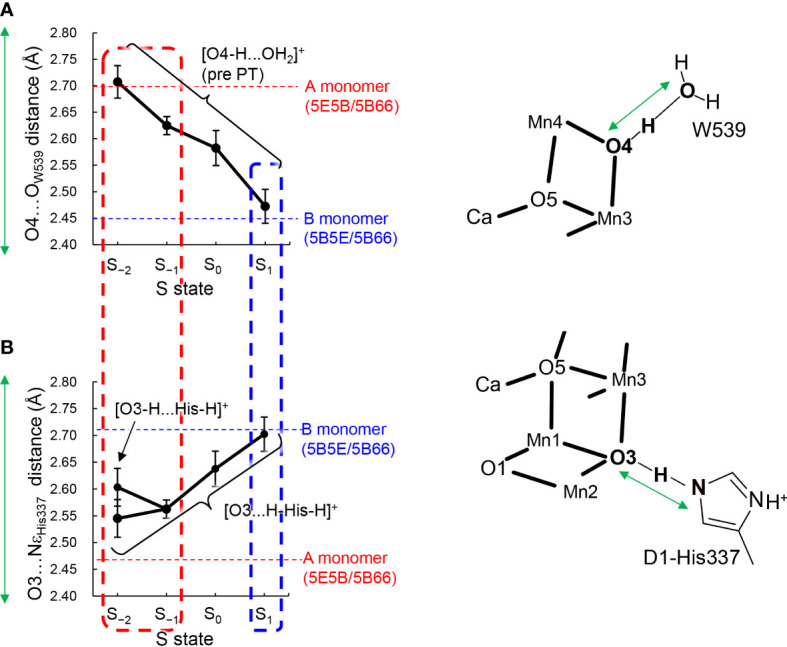
Dependence of calculated hydrogen-bond distances on the oxidation state (S state) of the Mn_4_CaO_5_ cluster in the QM/MM optimized structures. **(A)** The O4…O_W539_ distance in the protonated-O4 [O4-H…O_W539_H_2_]^+^ pattern, which was averaged over different protonation states of D1-His337 ([HN-His-NH]^+^ and [HN-His]^0^) and the two monomer units. **(B)** The O3…Nε_His337_ distance in doubly-protonated His337 ([H-His-H]^+^), which was averaged over different protonation states of O4 (the protonated and deprotonated O4 patterns) and the two monomer units. The dotted horizontal line indicates the averaged distance of the low-dose structures (PDB-IDs: 5E5B and 5B66) in the A (red) and B (blue) monomers shown in **Table 1**. The dotted box represents the best-fitted S state with the crystal-structure distances of the A (red; S_−1_ or S_−2_) and B (blue; S_1_) monomers. In S_−2_, the two conformations for the O3-His337 hydrogen bond (i.e., [O3-H…His-H]^+^ and [O3…H-His-H]^+^) were obtained. The error bars represent the standard deviations.

The QM/MM optimized geometries indicate that the O3…Nε_His337_ distance decreased as the Mn_4_CaO_5_ cluster is reduced from S_1_ to S_−1_ when D1-His337 is doubly-protonated ([HN-His-NH]^+^) (**Figure 3B**). In S_−2_, a proton can move from D1-His337 to O3 (i.e., [O3-H…His-H]^+^) and the O3…Nε_His337_ distance increases (2.63 Å) relative to S_−1_ (2.57 Å). In contrast, the O3…Nε_His337_ hydrogen bond (2.54 Å) is the shortest when D1-His337 is doubly-protonated (i.e., [O3…H-His-H]^+^). Proton transfer to O3 from D1-His337 during overreduction was previously reported in a DFT study (Galstyan et al., 2012). Proton transfer can easily occur in a short LBHB (~2.5 Å) (Ishikita and Saito, 2014). Accordingly, the short hydrogen-bond (2.46 and 2.48 Å) in the low-dose structure suggests that the LBHB exists in either S_−1_ or S_−2_. It should be noted that the O3…Nε_His337_ distance does not change upon oxidation when D1-His337 is singly-protonated ([HN-His]^0^) (**Figure S1a**).

The O4…O_W539_ distance decreases in the protonated O4 pattern ([O4-H … O_W539_H_2_]^+^) as the Mn_4_CaO_5_ cluster is oxidized from S_−2_ to S_2_ (**Figure 3A**). Thus, the O4…O_W539_ hydrogen bond in S_1_ is the shortest among all S states investigated, which is consistent with a previous QM/MM study showing that [O4-H…O_W539_H_2_]^+^ forms an LBHB in S_1_ (**Figure 1A**) (Saito et al., 2015).

The "standard" O3…Nε_His337_ and "short" O4…O_W539_ hydrogen bonds (**Figure 1B**) in the B monomer are best fitted to the case with doubly-protonated D1-His337 ([H-His-H]^+^) and deprotonated O4 ([O4…HO_W539_H]^0^) in S_1_ (**Figure 3**). In contrast, the short O3…Nε_His337_ and standard O4…O_W539_ hydrogen bonds in the A monomer are best fitted to the case with doubly-protonated D1-His337 ([H-His-H]^+^) and protonated O4 ([O4H...O_W539_H_2_]^+^) in S_−1_ or S_−2_ (**Figure 3**). Note that doubly-protonated D1-His337 was reported in a FTIR study (Nakamura and Noguchi, 2017). These results may indicate that the two monomer units are in different oxidation states (i.e., S_−2_ or S_−1_ for the A monomer and S_1_ for the B monomer) as speculated by Tanaka et al. (Tanaka et al., 2017). Notably, a machine learning study suggested that the oxidation state differs between monomer units (e.g., S_0_ for the A monomer and S_1_ for the B monomer in the 0.03 MGy structure (Amin, 2022).

**Figure 4:**
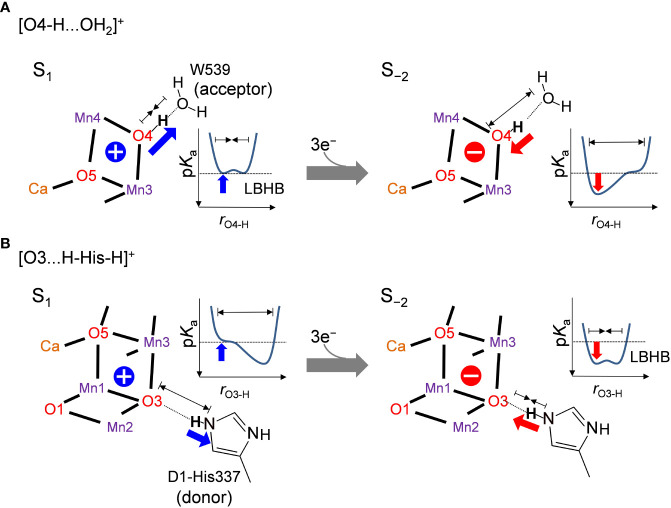
Relationship between the hydrogen bond distance and oxidation state of the Mn_4_CaO_5_ cluster when **(A)** O4 donates the hydrogen bond to W539 ([O4-H…O_W539_H_2_]^+^) and **(B)** O3 accepts the hydrogen bond from D1-His337 ([O3…H-His-H]^+^). Schematic illustrations of the hydrogen-bond potential-energy curves are shown. *r*_O4-H_ and *r*_O3-H_ represent the O4-H^+^ and O3-H^+^ distances, respectively.

The difference in the hydrogen-bond distance originates from the difference in the charge of the Mn_4_CaO_5_ cluster. The O4-H...O_W539_ hydrogen bond lengthens due to an increase in p*K*_a_ of the Mn_4_CaO_5_ cluster as the cluster is reduced (**Figure 4A**). Therefore, the short LBHB of [O4-H…O_W539_H_2_]^+^ in S_1_ is lost upon reduction, which results in a standard hydrogen bond (**Figure 4A**). The O3...His337 hydrogen bond shortens due to an increase in p*K*_a_ of the Mn_4_CaO_5_ cluster as the cluster is reduced (**Figure 4B**). Therefore, the short LBHB of [O3…H-His-H]^+^ in S_−2_ is lost upon oxidation, which results in a standard hydrogen bond (**Figure 4B**).

**Figure 5:**
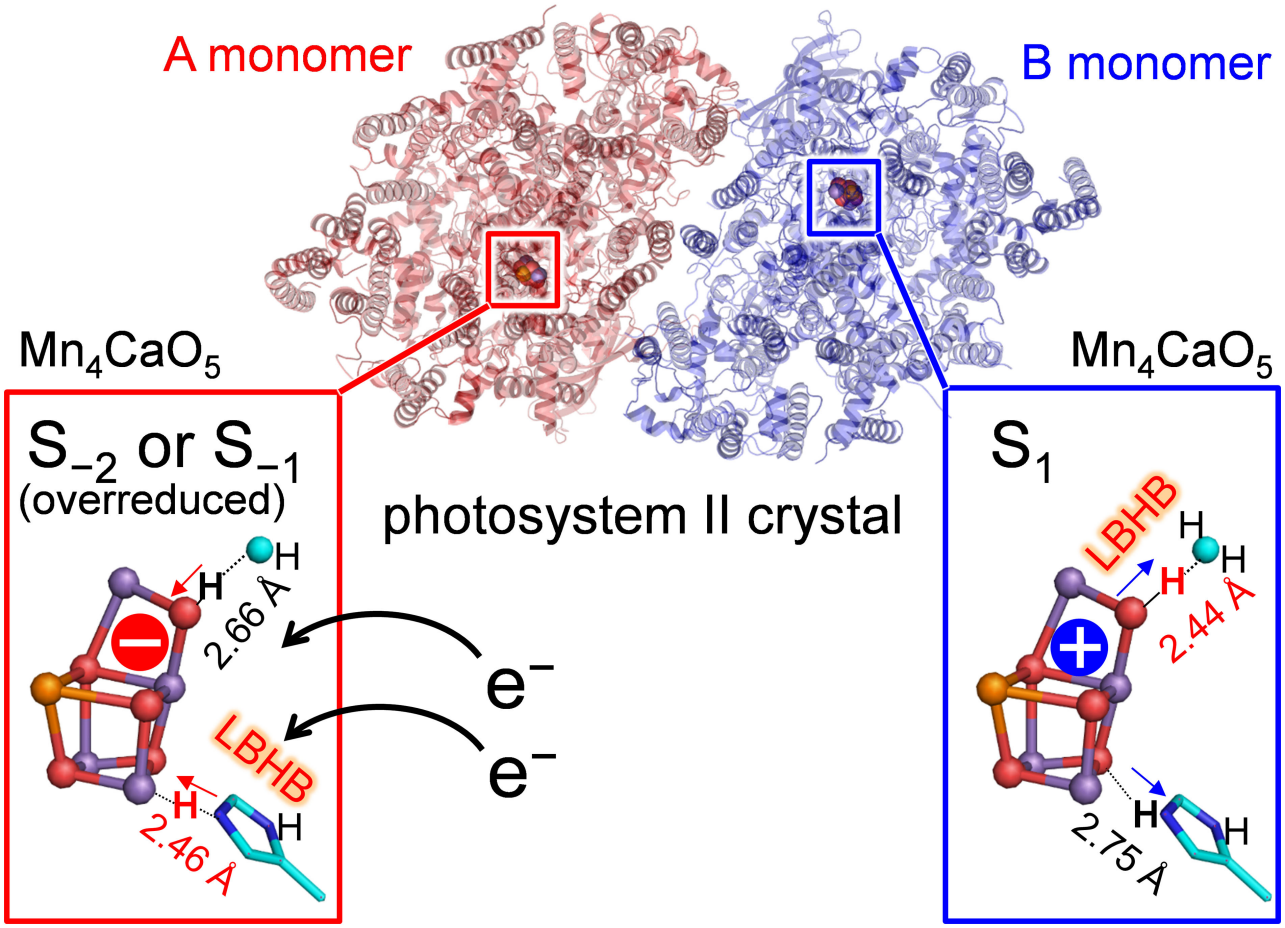
Different oxidation states and hydrogen-bond distances between the A and B monomers.

Thus, the [O4-H … O_W539_H_2_]^+^ hydrogen bond is longer in the overreduced state than that in S_1_, whereas the [O3…H-His-H]^+^ hydrogen bond is shorter in the overreduced state than that in the S_1_ state (**Figure 3**). In the low-dose structures, the O3…Nε_His337_ hydrogen bond is shorter in the A monomer than that in the B monomer, whereas the O4…O_W539_ hydrogen bond is longer in the A monomer than that in the B monomer (**Figure 2A**; **Table 1**). Thus, O4 is protonated and that the Mn_4_CaO_5_ cluster may be more reduced in the A monomer than in the B monomer (**Figure 5**).

These results suggest that A monomers are more damaged than B monomers in the crystal, although the X-ray dose received by both monomers should be identical. This may be explained by the difference in radical diffusion between the two monomer units. Water molecules can be involved in the free radical generation induced by X-ray (Ma et al., 2018). The free radical generation is particularly pronounced near metal cofactors (e.g., the Mn_4_CaO_5_ cluster), as they can absorb free electrons and be reduced (Beyerlein et al., 2018; Nass, 2019) as pointed out in theoretical studies on the XFEL structure (Mandal et al., 2021). Therefore, the arrangement of water molecules near the Mn_4_CaO_5_ cluster may be a factor for the difference in the radiation damage. In the PSII crystal structure (Umena et al., 2011; Suga et al., 2015), the number and arrangement of water molecules of the O4 channel differ between monomer units [see Table S1 in (Sakashita et al., 2020)]. The structural difference might also be due to the difference in crystal packing (Tanaka et al., 2017).

In the Mn_4_CaO_5_ cluster of the low-dose structure, the Mn4-O4 distances are 1.87 and 2.27 Å and the Mn3-O3 distances are 2.27 and 1.96 Å in the A and B monomers, respectively (Tanaka et al., 2017). The calculated geometries did not reproduce the differences (**Table S3**).

In summary, the difference in the hydrogen-bond distance between the two homogeneous (A and B) monomer units in the low-dose structure originates from the difference in the D1-His337/O4 protonation state and the Mn_4_CaO_5_ oxidation state. The short O4-W539 hydrogen bond in the B monomer can be reproduced when O4 is protonated in S_1_ (**Table 1** and **Figure 3A**). The short O3-His337 hydrogen bond in the A monomer indicates that the LBHB forms between O3 and doubly-protonated D1-His337 in S_−1_ or S_−2_ (**Table 1** and **Figure 3B**). These results suggest that the Mn_4_CaO_5_ oxidation state differs between the two monomer units (i.e., S_−1_ or S_−2_ for the A monomer and S_1_ for the B monomer; **Figure 5**).

## Data availability statement

The raw data supporting the conclusions of this article will be made available by the authors, without undue reservation.

## Author contributions

HI designed research. KS and SN performed research. KS and HI wrote the manuscript. All authors contributed to the article and approved the submitted version.

## References

[B1] Ames W. Pantazis D. A. Krewald V. Cox N. Messinger J. Lubitz W.. (2011). Theoretical evaluation of structural models of the S_2_ state in the oxygen evolving complex of photosystem II: Protonation states and magnetic interactions. J. Am. Chem. Soc. 133 (49), 19743–19757. doi: 10.1021/ja2041805 22092013

[B2] Amin M. (2022). Predicting the oxidation states of Mn ions in the oxygen-evolving complex of photosystem II using supervised and unsupervised machine learning. Photosyn. Res. doi: 10.1007/s11120-022-00941-8 PMC10070209 35896927

[B3] Askerka M. Vinyard D. J. Wang J. Brudvig G. W. Batista V. S. (2015). Analysis of the radiation-damage-free X-ray structure of photosystem II in light of EXAFS and QM/MM data. Biochemistry 54 (9), 1713–1716. doi: 10.1021/acs.biochem.5b00089 25710258

[B4] Beyerlein K. R. Jonsson H. O. Alonso-Mori R. Aquila A. Barty S. Barty A.. (2018). Ultrafast nonthermal heating of water initiated by an X-ray free-electron laser. Proc. Natl. Acad. Sci. U.S.A. 115 (22), 5652–5657. doi: 10.1073/pnas.1711220115 29760050 PMC5984484

[B5] Brooks B. R. Bruccoleri R. E. Olafson B. D. States D. J. Swaminathan S. Karplus M. (1983). CHARMM: A program for macromolecular energy minimization and dynamics calculations. J. Comput. Chem. 4 (2), 187–217. doi: 10.1002/jcc.540040211

[B6] Cardona T. Rutherford A. W. (2019). Evolution of photochemical reaction centres: More twists? Trends Plant Sci. 24 (11), 1008–1021. doi: 10.1016/j.tplants.2019.06.016 31351761

[B7] Galstyan A. Robertazzi A. Knapp E. W. (2012). Oxygen-evolving Mn cluster in photosystem II: The protonation pattern and oxidation state in the high-resolution crystal structure. J. Am. Chem. Soc. 134 (17), 7442–7449. doi: 10.1021/ja300254n 22489676

[B8] Glockner C. Kern J. Broser M. Zouni A. Yachandra V. Yano J. (2013). Structural changes of the oxygen-evolving complex in photosystem II during the catalytic cycle. J. Biol. Chem. 288 (31), 22607–22620. doi: 10.1074/jbc.M113.476622 23766513 PMC3829347

[B9] Grabolle M. Haumann M. Muller C. Liebisch P. Dau H. (2006). Rapid loss of structural motifs in the manganese complex of oxygenic photosynthesis by X-ray irradiation at 10-300 K. J. Biol. Chem. 281 (8), 4580–4588. doi: 10.1074/jbc.M509724200 16352605

[B10] Ikeda T. Saito K. Hasegawa R. Ishikita H. (2017). The existence of an isolated hydronium ion in the interior of proteins. Angew. Chem, Int. Ed. 56 (31), 9151–9154. doi: 10.1002/anie.201705512 PMC5575531 28613440

[B11] Ishikita H. Saito K. (2014). Proton transfer reactions and hydrogen-bond networks in protein environments. J. R. Soc. Interface 11 (91), 20130518. doi: 10.1098/rsif.2013.0518 24284891 PMC3869154

[B12] Ishikita H. Saenger W. Loll B. Biesiadka J. Knapp E. W.. (2006). Energetics of a possible proton exit pathway for water oxidation in photosystem II. Biochemistry 45 (7), 2063–2071. doi: 10.1021/bi051615h 16475795

[B13] Isobe H. Shoji M. Yamanaka S. Umena Y. Kawakami K. Kamiya N.. (2012). Theoretical illumination of water-inserted structures of the CaMn_4_O_5_ cluster in the S_2_ and S_3_ states of oxygen-evolving complex of photosystem II: Full geometry optimizations by B3LYP hybrid density functional. Dalton Trans. 41 (44), 13727–13740. doi: 10.1039/c2dt31420g 23037319

[B14] Kawashima K. Saito K. Ishikita H. (2018). Mechanism of radical formation in the H-bond network of D1-Asn298 in photosystem II. Biochemistry 57 (33), 4997–5004. doi: 10.1021/acs.biochem.8b00574 30015472

[B15] Krewald V. Retegan M. Cox N. Messinger J. Lubitz W. DeBeer S.. (2015). Metal oxidation states in biological water splitting. Chem. Sci. 6, 1676–1695. doi: 10.1039/C4SC03720K 29308133 PMC5639794

[B16] Lohmiller T. Krewald V. Sedoud A. Rutherford A. W. Neese F. Lubitz W.. (2017). The first state in the catalytic cycle of the water-oxidizing enzyme: Identification of a water-derived μ-hydroxo bridge. J. Am. Chem. Soc. 139 (41), 14412–14424. doi: 10.1021/jacs.7b05263 28921983

[B17] Luber S. Rivalta I. Umena Y. Kawakami K. Shen J.-R. Kamiya N.. (2011). S_1_-state model of the O_2_-evolving complex of photosystem II. Biochemistry 50, 6308–6311. doi: 10.1021/bi200681q 21678908 PMC3139771

[B18] Ma J. Wang F. R. Mostafavi M. (2018). Ultrafast chemistry of water radical cation, H_2_O^·+^, in aqueous solutions. Molecules 23 (2), 244. doi: 10.3390/molecules23020244 29373497 PMC6017428

[B19] MacKerell A. D. Bashford D. Bellott M. Dunbrack R. L. Evanseck J. D. Field M. J.. (1998). All-atom empirical potential for molecular modeling and dynamics studies of proteins. J. Phys. Chem. B 102 (18), 3586–3616. doi: 10.1021/jp973084f 24889800

[B20] Mandal M. Saito K. Ishikita H. (2021). Two distinct oxygen-radical conformations in the X‑ray free electron laser structures of photosystem II. J. Phys. Chem. Lett. 12 (16), 4032–4037. 33881870 10.1021/acs.jpclett.1c00814

[B21] Mandal M. Kawashima K. Saito K. Ishikita H. (2020). Redox potential of the oxygen-evolving complex in the electron transfer cascade of photosystem II. J. Phys. Chem. Lett. 11 (1), 249–255. doi: 10.1021/acs.jpclett.9b02831 31729876

[B22] Nakamura S. Noguchi T. (2017). Infrared determination of the protonation state of a key histidine residue in the photosynthetic water oxidizing center. J. Am. Chem. Soc. 139 (27), 9364–9375. doi: 10.1021/jacs.7b04924 28635275

[B23] Nass K. (2019). Radiation damage in protein crystallography at X-ray free-electron lasers. Acta Cryst. D75, 211–218. doi: 10.1107/S2059798319000317 PMC6400258 30821709

[B24] QSite (2012). Schrödinger, LLC (New York). version 5.8.

[B25] Saito K. Ishikita H. (2019). Mechanism of protonation of the over-reduced Mn_4_CaO_5_ cluster in photosystem II. Biochim. Biophys. Acta Bioenerg 1860 (10), 148059. doi: 10.1016/j.bbabio.2019.148059 31394097

[B26] Saito K. Rutherford A. W. Ishikita H. (2015). Energetics of proton release on the first oxidation step in the water-oxidizing enzyme. Nat. Commun. 6, 8488. doi: 10.1038/ncomms9488 26442814 PMC4617610

[B27] Saito K. Shen J.-R. Ishida T. Ishikita H. (2011). Short hydrogen-bond between redox-active tyrosine Y_Z_ and D1-His190 in the photosystem II crystal structure. Biochemistry 50, 9836–9844. doi: 10.1021/bi201366j 21972783

[B28] Sakashita N. Ishikita H. Saito K. (2020). Rigidly hydrogen-bonded water molecules facilitate proton transfer in photosystem II. Phys. Chem. Chem. Phys. 22 (28), 15831–15841. doi: 10.1039/d0cp00295j 32613215

[B29] Shen J. R. (2015). The structure of photosystem II and the mechanism of water oxidation in photosynthesis. Annu. Rev. Plant Biol. 66, 23–48. doi: 10.1146/annurev-arplant-050312-120129 25746448

[B30] Shimizu T. Sugiura M. Noguchi T. (2018). Mechanism of proton-coupled electron transfer in the S_0_ to S_1_ transition of photosynthetic water oxidation as revealed by time-resolved infrared spectroscopy. J. Phys. Chem. B 122 (41), 9460–9470. doi: 10.1021/acs.jpcb.8b07455 30251857

[B31] Siegbahn P. E. (2013). Water oxidation mechanism in photosystem II, including oxidations, proton release pathways, O-O bond formation and O_2_ release. Biochim. Biophys. Acta 1827 (8-9), 1003–1019. doi: 10.1016/j.bbabio.2012.10.006 23103385

[B32] Suga M. Akita F. Hirata K. Ueno G. Murakami H. Nakajima Y.. (2015). Native structure of photosystem II at 1.95 Å resolution viewed by femtosecond X-ray pulses. Nature 517, 99–103. doi: 10.1038/nature13991 25470056

[B33] Suzuki H. Sugiura M. Noguchi T. (2005). pH dependence of the flash-induced S-state transitions in the oxygen-evolving center of photosystem II from *Thermosynechoccocus elongatus* as revealed by Fourier transform infrared spectroscopy. Biochemistry 44, 1708–1718. doi: 10.1021/bi0483312 15683255

[B34] Takaoka T. Sakashita N. Saito K. Ishikita H. (2016). p*K* _a_ of a proton-conducting water chain in photosystem II. J. Phys. Chem. Lett. 7 (10), 1925–1932. doi: 10.1021/acs.jpclett.6b00656 27128410

[B35] Tanaka A. Fukushima Y. Kamiya N. (2017). Two different structures of the oxygen-evolving complex in the same polypeptide frameworks of photosystem II. J. Am. Chem. Soc. 139 (5), 1718–1721. doi: 10.1021/jacs.6b09666 28102667

[B36] Umena Y. Kawakami K. Shen J.-R. Kamiya N. (2011). Crystal structure of oxygen-evolving photosystem II at a resolution of 1.9 Å. Nature 473 (7345), 55–65. doi: 10.1038/nature09913 21499260

[B37] Vacek G. Perry J. K. Langlois J. M. (1999). Advanced initial-guess algorithm for self-consistent-field calculations on organometallic systems. Chem. Phys. Lett. 310, 189–194. doi: 10.1016/S0009-2614(99)00722-8

[B38] Yamamoto M. Nakamura S. Noguchi T. (2020). Protonation structure of the photosynthetic water oxidizing complex in the S_0_ state as revealed by normal mode analysis using quantum mechanics/molecular mechanics calculations. Phys. Chem. Chem. Phys. 22 (42), 24213–24225. doi: 10.1039/d0cp04079g 33084674

[B39] Yano J. Kern J. Irrgang K. D. Latimer M. J. Bergmann U. Glatzel P.. (2005). X-Ray damage to the Mn_4_Ca complex in single crystals of photosystem II: A case study for metalloprotein crystallography. Proc. Natl. Acad. Sci. U.S.A. 102 (34), 12047–12052. doi: 10.1073/pnas.0505207102 16103362 PMC1186027

