# Supplementary material for: Identification of the protonation and oxidation states of the oxygen-evolving complex in the low-dose X-ray crystal structure of photosystem II
Source: Front Plant Sci. 2023 Mar 16;14:1029674. doi: 10.3389/fpls.2023.1029674 (PMC10061019; doi:10.3389/fpls.2023.1029674)
Supplement: Supplementary file 1 [file DataSheet_1.pdf]

Supporting information for:

Identification of the protonation and oxidation states of the oxygen-evolving complex in the low-dose X-ray crystal structure of photosystem II

Keisuke Saito*[1,2], Shu Nakao [1], and Hiroshi Ishikita*[1,2]

1) Department of Applied Chemistry, The University of Tokyo, 7-3-1 Hongo, Bunkyo-ku, Tokyo 113-8654, Japan

2) Research Center for Advanced Science and Technology, The University of Tokyo, 4-6-1 Komaba, Meguro-ku, Tokyo 153-8904, Japan

CORRESPONDING AUTHOR:

Keisuke Saito, **E-mail:** ksaito@appchem.t.u-tokyo.ac.jp

Hiroshi Ishikita, **E-mail:** hiro@appchem.t.u-tokyo.ac.jp

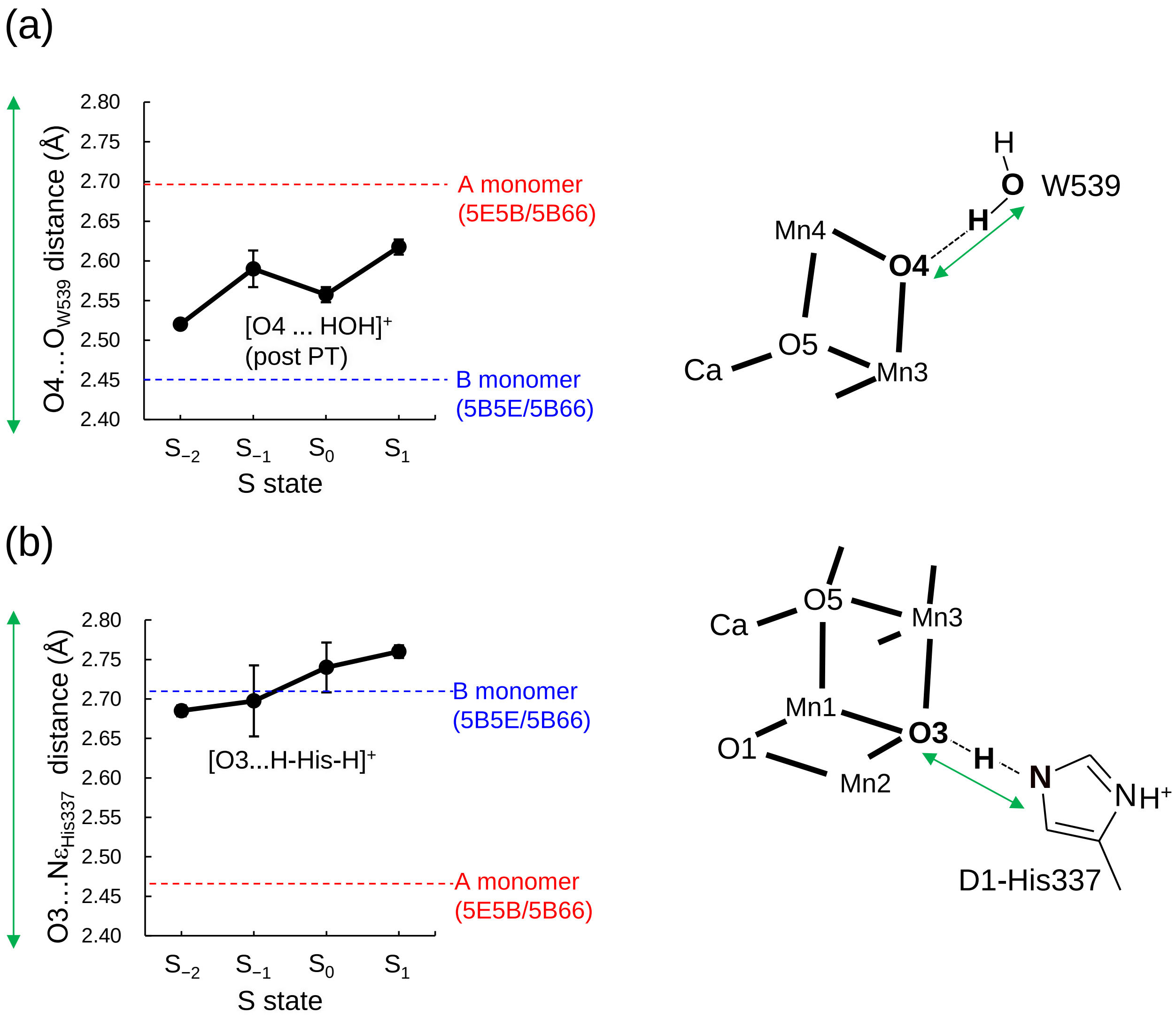

Figure S1 Dependence of calculated hydrogen-bond distances on the oxidation state (S state) of the Mn_4CaO_5 in the QM/MM optimized structures. (a) The O4…O_{W539} distance in the protonated-O4 $[O4...H\text{-}O_{W539}H]^+$ pattern, which was averaged over different protonation states of D1-His337 ($[HN\text{-}His\text{-}NH]^+$ and $[HN\text{-}His]^0$) and the two monomer units. (b) The O3…$N\varepsilon_{His337}$ distance in the singly-protonated His337 ($[H\text{-}His]^0$), which was averaged over different protonation states of O4 (the protonated O4 and deprotonated O4 patterns) and the two monomers. The dotted horizontal line indicates the averaged distance of the low-dose structures (PDB IDs: 5E5B and 5B66) in the A (red) and B (blue) monomers shown in Table 1.

Table S1. Hydrogen bond distances for the low dose structure and QM/MM-optimized structures (in Å) in the anti ferromagnetically coupled (i.e., low spin) case. Short distances (< 2.5 Å for the O-O distance and < 2.55 Å for the O-N distance) are in bold. —, not applicable.

**Unit** [a]				**Spin configration**	**A**		**B**	
**Structure**		**D1-His337**	**O4-W539**	**(Mn1,Mn2,Mn3,Mn4)**	**O4…O_{W539}**	**O3…$N\varepsilon_{His337}$**	**O4…O_{W539}**	**O3…$N\varepsilon_{His337}$**
**Crystal**								
Low-dose								
5B5E (0.03 MGy)					2.66	**2.46**	**2.44**	2.75
5B66 (0.12 MGy)					2.71	**2.48**	**2.45**	2.74
**QM/MM**								
(Standard oxidation state)								
Low-dose (5B5E)	S_0	$[H\text{-}His\text{-}H]^+$	$[O4...H...OH_2]^+$ [b]	(↑↓↑↓)	2.59	2.67	2.55	2.69
		$[His\text{-}H]^0$		(↑↓↑↓)	2.60	2.75	2.56	2.75
		$[H\text{-}His\text{-}H]^+$	$[O4...HOH]^0$ [c]	(↑↑↓↓)	2.56	2.62	2.57	2.62
		$[His\text{-}H]^0$		(↑↑↓↓)	2.56	2.76	2.55	2.74
	S_1	$[H\text{-}His\text{-}H]^+$	$[O4...H...OH_2]^+$ [d]	(↑↑↓↓)	**2.48**	2.72	**2.44**	2.75
		$[His\text{-}H]^0$		(↑↓↑↓)	**2.49**	2.78	**2.45**	2.80
		$[H\text{-}His\text{-}H]^+$	$[O4...HOH]^0$ [d]	(↑↑↓↓)	2.63	2.67	2.62	2.69
		$[His\text{-}H]^0$		(↑↓↑↓)	2.59	2.77	2.61	2.73
(Over-reduced state)								
Low-dose (5B5E)	S_{-1}	$[H\text{-}His\text{-}H]^+$	$[O4...H...OH_2]^+$ [e]	(↑↑↓↓)	2.64	2.59	2.61	2.59
		$[His\text{-}H]^0$		(↑↑↓↓)	2.64	2.74	2.61	2.73
		$[H\text{-}His\text{-}H]^+$	$[O4...HOH]^0$ [e]	(↑↑↓↓)	2.57	2.55	2.56	2.55
		$[His\text{-}H]^0$		(↑↑↓↓)	2.60	2.72	2.59	2.71
	S_{-2}	$[H\text{-}His\text{-}H]^+$	$[O4...H...OH_2]^+$ [f]	(↓↑↓↑)	2.70	2.60	2.67	2.57
		$[His\text{-}H]^0$		(↓↑↓↑)	2.65	2.70	2.65	2.73
		$[H\text{-}His\text{-}H]^+$	$[O4...HOH]^0$ [f]	(↓↑↓↑)	2.52	2.64	—[g]	—[g]
		$[His\text{-}H]^0$			—[g]	—[g]	—[g]	—[g]

[a] PSII monomer unit ID in the PSII dimer. [b] (Mn1, Mn2, Mn3, Mn4) = (III, IV, III, III). [c] (Mn1, Mn2, Mn3, Mn4) = (III, III, IV, III). [d] (Mn1, Mn2, Mn3, Mn4) = (III, IV, IV, III). [e] (Mn1, Mn2, Mn3, Mn4) = (III, III, III, III). [f] (Mn1, Mn2, Mn3, Mn4) = (III, III, III, II). [g] The S_{-2} state could not be obtained because of an internal electron transfer from Mn4 to D1-Asn298.

Table S2. Calculated Mulliken spin populations in the ferromagnetically coupled case.

Unit [a]					A				B			
**Structure**		**D1-His337**	**O4-W539**	**Valence**	**Mn1**	**Mn2**	**Mn3**	**Mn4**	**Mn1**	**Mn2**	**Mn3**	**Mn4**
**QM/MM**												
(standard oxidation state)												
Low-dose (5B5E) [a]	S_0	[H-His-H]$^+$	[O4...H...OH_2]$^+$	(III,IV,III,III)	3.830	2.960	3.837	3.842	3.835	2.956	3.839	3.844
		[His-H]^0		(III,IV,III,III)	3.824	2.974	3.840	3.838	3.830	2.970	3.841	3.839
		[H-His-H]$^+$	[O4...HOH]^0	(III,III,IV,III)	3.847	3.857	2.963	3.863	3.842	3.847	2.959	3.857
		[His-H]^0		(III,III,IV,III)	3.842	3.864	2.962	3.852	3.834	3.857	2.957	3.850
	S_1	[H-His-H]$^+$	[O4...H...OH_2]$^+$	(III,IV,IV,III)	3.829	2.966	2.970	3.871	3.832	2.965	2.968	3.870
		[His-H]^0		(III,IV,IV,III)	3.823	2.972	2.976	3.876	3.828	2.971	2.975	3.874
		[H-His-H]$^+$	[O4...HOH]^0	(III,IV,IV,III)	3.842	2.943	2.879	3.862	3.840	2.942	2.864	3.875
		[His-H]^0		(III,IV,IV,III)	3.838	2.957	2.907	3.853	3.842	2.953	2.880	3.865
(over-reduced state)												
Low-dose (5B5E) [a]	S_{-1}	[H-His-H]$^+$	[O4...H...OH_2]$^+$	(III,III,III,III)	3.823	3.881	3.856	3.832	3.818	3.867	3.857	3.835
		[His-H]^0		(III,III,III,III)	3.824	3.889	3.836	3.822	3.812	3.875	3.851	3.824
		[H-His-H]$^+$	[O4...HOH]^0	(III,III,III,III)	3.834	3.872	3.801	3.849	3.823	3.856	3.801	3.863
		[His-H]^0		(III,III,III,III)	3.856	3.853	3.792	3.859	3.843	3.841	3.796	3.876
	S_{-2}	[H-His-H]$^+$	[O4...H...OH_2]$^+$	(III,III,III,II)	3.822	3.880	3.825	4.803	3.818	3.857	3.834	4.803
		[His-H]^0		(III,III,III,II)	3.808	3.881	3.804	4.802	3.734	3.857	3.831	4.692
		[H-His-H]$^+$	[O4...HOH]^0	(III,III,III,II)	3.808	3.881	3.804	4.802	—	—	—	—
		[His-H]^0		—	—	—	—	—	—	—	—	—

Table S3. Mn3-O3 and Mn4-O4 distances for the low dose structure and QM/MM-optimized structures (in Å) in the ferromagnetically coupled case.

**Unit [a]**				**A**		**B**	
**Structure**		**D1-His337**	**O4-W539**	**Mn3-O3**	**Mn4-O4**	**Mn3-O3**	**Mn4-O4**
**Crystal**							
Low-dose							
5B5E (0.03 MGy)				2.27	1.87	1.96	2.07
5B66 (0.12 MGy)				2.18	1.84	1.95	2.10
**QM/MM**							
(standard oxidation state)							
Low-dose (5B5E) [a]	S_0	[H-His-H]$^{+}$	[O4...H...OH$_2$]$^{+}$	2.01	2.03	2.02	2.02
		[His-H]^{0}		1.97	2.04	1.98	2.03
		[H-His-H]$^{+}$	[O4...HOH]^{0}	1.91	1.80	1.92	1.79
		[His-H]^{0}		1.87	1.79	1.88	1.79
	S_1	[H-His-H]$^{+}$	[O4...H...OH$_2$]$^{+}$	1.95	2.07	1.96	2.03
		[His-H]^{0}		1.92	2.08	1.93	2.06
		[H-His-H]$^{+}$	[O4...HOH]^{0}	1.96	1.80	1.97	1.80
		[His-H]^{0}		1.94	1.79	1.94	1.79
(over-reduced state)							
Low-dose (5B5E) [a]	S_{-1}	[H-His-H]$^{+}$	[O4...H...OH$_2$]$^{+}$	1.93	1.99	1.94	1.99
		[His-H]^{0}		1.88	1.95	1.89	1.98
		[H-His-H]$^{+}$	[O4...HOH]^{0}	1.97	1.84	1.96	1.83
		[His-H]^{0}		1.94	1.82	1.93	1.82
	S_{-2}	[H-His-H]$^{+}$	[O4...H...OH$_2$]$^{+}$	1.97	2.19	1.97	2.20
		[His-H]^{0}		1.89	2.18	1.89	2.17
		[H-His-H]$^{+}$	[O4...HOH]^{0}	2.00	2.13	—[g]	—[g]
		[His-H]^{0}		—[g]	—[g]	—[g]	—[g]
